# Different frequency control mechanisms and the exploitation of frequency space in passerines

**DOI:** 10.1002/ece3.7510

**Published:** 2021-04-07

**Authors:** Franz Goller, Jay Love, Gabriel Mindlin

**Affiliations:** ^1^ School of Biological Sciences University of Utah Salt Lake City UT USA; ^2^ Institute for Zoophysiology University of Münster Münster Germany; ^3^ Department of Physics University of Buenos Aires Buenos Aires Argentina

**Keywords:** birdsong, control mechanisms, exploitation of frequency space, frequency modulation, frequency space, passerines, song evolution, song frequency, suboscine/oscine, tension control, vocal repertoire

## Abstract

1. Birdsong is used in reproductive context and, consequently, has been shaped by strong natural and sexual selection. The acoustic performance includes a multitude of acoustic and temporal characteristics that are thought to honestly reveal the quality of the singing individual.
2. One major song feature is frequency and its modulation. Sound frequency can be actively controlled, but the control mechanisms differ between different groups. Two described mechanisms are pressure‐driven frequency changes in suboscines and control by syringeal muscles in oscines.
3. To test to what degree these different control mechanisms enhance or limit the exploitation of frequency space by individual species and families, we compared the use of frequency space by tyrannid suboscines and emberizid/passerellid oscines.
4. We find that despite the different control mechanisms, the songs of species in both groups can contain broad frequency ranges and rapid and sustained frequency modulation (FM). The maximal values for these parameters are slightly higher in oscines.
5. Furthermore, the mean frequency range of song syllables is substantially larger in oscines than suboscines. Species within each family group collectively exploit equally broadly the available frequency space.
6. The narrower individual frequency ranges of suboscines likely indicate morphological specialization for particular frequencies, whereas muscular control of frequency facilitated broader exploitation of frequency space by individual oscine species.

## Graphical abstract

Suboscines and oscines achieve similar frequency modulation rates despite different frequency control mechanisms. As clades, both achieve similar exploitation of frequency space, but individual species of the vocal learning oscines encompass a broader frequency range in their vocal repertoires than suboscines.

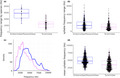

## 1 INTRODUCTION

Behaviors linked to reproduction play a pivotal role in evolution. Signaling behaviors are critically important in courtship (mate attraction and mate choice) and in defending resources for reproduction (male–male competition). Frequently, vocal signals are a part of these displays, and specifically in birds, acoustic signals are widely used and often constitute complex acoustic sequences, songs (e.g., Bradbury & Vehrencamp, 2011; Searcy & Nowicki, 2005). Strong sexual and natural selection forces are thought to act on song, driving evolutionary change that might lead to reproductive isolation, thus affecting speciation events (Andersson, 1994; Searcy & Andersson, 1986).

Acoustic performance can be assessed using many acoustic and temporal parameters. One such parameter is sound frequency, which gives rise to multiple, perceptually salient acoustic attributes. Among these is diversity within the vocal repertoire, which may be manifested in a broad overall range and/or range of individual syllables by modulation of frequency. The latter has been studied widely in the context of a trade‐off between frequency range of individual syllables and syllable repetition rate (for review, e.g., Podos & Sung, 2020).

In birds, vocal behavior is produced by airflow‐induced vibrations of oscillating tissues (labia or membranes) in the uniquely avian sound‐producing organ, the syrinx. In Passeriformes, the syrinx contains two sound sources. The respiratory system generates the airstream for phonation, and active adjustments in the syrinx can influence aerodynamic conditions and other parameters for sound production (e.g., Düring & Elemans, 2016; Goller, 2017; Riede & Goller, 2010). Aside from oscines, very little is known about how active syringeal control contributes to the acoustic characteristics of vocal repertoires.

Sound frequency can be controlled by different mechanisms (Goller & Riede, 2013). In oscines, frequency is mainly controlled by action of syringeal muscles, which adjust the tension of the vibrating labia. The activity of the ventral syringeal muscle is closely correlated with fundamental frequency of ipsilaterally generated sound (e.g., Goller & Riede, 2013; Goller & Suthers, 1996). In addition, other syringeal muscles contribute indirectly to frequency control (e.g., Döppler, et al., 2018; Döppler, et al., 2018; Méndez & Goller, 2020; Srivastava et al., 2015). Thus, if syringeal muscles are denervated, sound frequency drops markedly (e.g., Riede et al., 2010; Seller, 1979; Suthers et al., 2004; Williams et al., 1992). The driving air sac pressure also affects the tension of the vibrating labia (Goller & Riede, 2013), but pressure‐driven changes in fundamental frequency are small compared with muscle‐induced changes (e.g., Riede et al., 2010).

In contrast, in tyrannid species syringeal muscle activity may not be directly involved in regulating labial tension. Denervation does not markedly alter the trajectory of fundamental frequency in the songs of the three investigated species (great kiskadee, *Pitangus sulphuratus*, Amador et al., 2008; Döppler et al., 2020; Western kingbird, *Tyrannus verticalis*, Garcia and Goller, unpublished; Western wood pewee, *Contopus sordidulus*, Peltier and Goller, unpublished). Instead, sound frequency shows a tight correlation with the driving air sac pressure, suggesting that pressure conditions affect the tension of the vibrating labia and thus control FM (Amador et al., 2008). In tyrannids, changes in driving air sac pressure lead to substantial changes in sound frequency. Why pressure changes affect labial tension so differently in these groups is unknown.

This difference in frequency control mechanisms between suboscines and oscines leads to the question to what degree each group makes use of the physiologically possible (both production and perception) and ecologically available frequency range, here termed frequency space, in its environment. Does the frequency control mechanism limit frequency ranges of vocal repertoires or FM rates differently for the two groups? Answers to this question will give insight into evolutionary aspects such as how song may have contributed to speciation events and how physiological constraints may affect the use of acoustic space by different taxa.

Here, we explore the use of frequency space in passerine families. To make use of the close taxonomic relationship among passerine groups, which underwent remarkable radiations over the last 65 million years (e.g., Mitchell et al., 2016), we compared songs from the suboscine Tyrannidae (>300 spp.) and the oscine Emberizidae and Passerellidae (44 Old World and 139 New World spp., respectively). Because the species of the family Passerellidae show broad overlap in habitat with tyrannids, this selection allowed us to partially exclude potential ecological differences between the compared groups. The results show that both frequency control mechanisms can give rise to remarkably broad frequency ranges. The main difference was found in frequency range of vocal repertoires, which is attributable to the different control mechanisms. Despite important differences emerging from the respective vocal control systems between tyrannid and emberizid/passerellid species, songs in both groups evolved to make use of the available frequency space.

## 2 MATERIALS AND METHODS

### 2.1 Recordings

Songs were selected from the Xeno‐canto database (see data sets for species list) from species in the families Tyrannidae and Emberizidae/Passerellidae (the family Passerellidae was recognized as separate from Emberizidae during the course of this investigation). For each species, one song file was selected from each of 3–4 individuals. Recordings were inspected to assure a good signal‐to‐noise ratio and to minimize overlap with noise or acoustic signals from other animals. Depending on availability, we selected songs from as many genera as possible within each family group, resulting in data for 98 tyrannid species and 36 emberizid/passerellid (10/26) species (Appendix S1).

For each individual, at least 3 syllables were measured for each visually identified syllable type. In syllables that showed gradual variation, up to 10 syllables were measured, such that the full range of frequencies could be captured. In oscines with large song repertoires, all songs on each recording were inspected, so that all unique syllable types could be included in the frequency measurements.

### 2.2 Body mass

Body mass data for each species were taken from Dunning (2008). If multiple means were listed for different populations, we used the one that was closest geographically to the recording sites of songs analyzed for this study.

### 2.3 Song analysis

Song recordings were analyzed using Praat (v. 6.0.33) software. Each syllable was selected, and fundamental frequency was extracted (pitch tracking function) every 0.5 ms. For each tracking, the pitch tracking was visually verified, and parameters in the function were adjusted where needed to avoid erroneous frequency jumps or omitted sections. Frequency and time data were exported into spreadsheets, and various frequency statistics were determined for each syllable (e.g., minimal, maximal, and mean frequency). In addition, we calculated FM rates by using an averaging system over three different time steps. The mean of frequency changes over a 1, 2, and 3 time step period was calculated to avoid misleading extreme values from one time step (0.5 ms), which might have resulted from jumps in the pitch tracking series. This analysis resulted in data for 650 individual syllables in Emberizidae/Passerellidae and 655 syllables in Tyrannidae.

### 2.4 Phylogeny

From the online resource birdtree.org (Jetz et al., 2012), we downloaded 100 trees containing all 98 tyrannid and 36 emberizid/passerellid species in our sample drawn from the full Hackett backbone. Using the phytools package (v0.6‐44, Revell, 2012) in R, we then created an average tree, which we used for accounting for the effects of phylogeny in statistical tests, and two clade‐specific trees that were used to measure phylogenetic signal of traits.

### 2.5 Data analysis

All statistics were done in R v3.5.1 (R Core Team, 2018). Comparisons of features between clades were conducted with ANOVAs and Welch's two‐sample *t* tests. To investigate the relationship between FM rate and syllable duration, as below, we used 90th quantile linear regressions of log‐transformed data (“rq” function from quantreg package in R; Koenker, 2018). To determine the relationship between body mass and frequency, we used phylogenetically controlled generalized least‐squares (PGLS) models assuming a phylogenetic signal‐controlled correlation structure (“corPagel” function from the ape package in R to establish correlation structures considering our clade‐specific phylogenies, which we then used as the correlation structures in generalized least‐squares models obtained with the “gls” function from nlme package in R; Paradis & Schliep, 2018; Pinheiro et al., 2019). To test for phylogenetic signal of traits, we estimated both Blomberg's *K* and Pagel's lambda with the “phylosig” function from the phytools package in R (Revell, 2012). These two metrics use different methods to measure the degree to which related species share traits; Pagel's lambda is a correlation scaling factor, while Blomberg's *K* is a variance ratio (Blomberg et al., 2003; Münkemüller et al., 2012; Pagel, 1999). We used both methods and checked for agreement between them because the two metrics may not report the same level of phylogenetic signal for the same data set (Münkemüller et al., 2012). Phylogenetic trees with continuous trait mapping were plotted with the “contMap” function from the phytools package in R (Revell, 2012).

## 3 RESULTS

**FIGURE 1:**
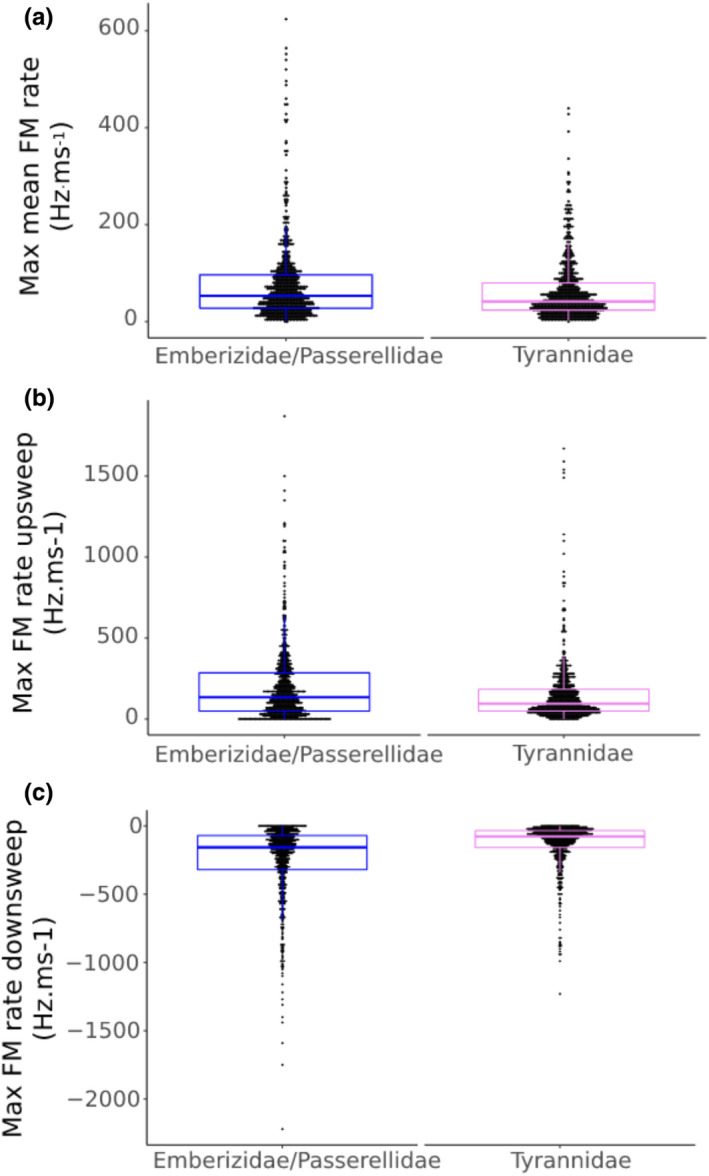
(a) Maximal mean FM rate (Hz/ms) is slightly higher in Emberizidae/Passerellidae (78.30 Hz/ms) than Tyrannidae (62.35 Hz/ms) (ANOVA: *F* = 14.55, *df* = 1,304, *p* = .00015), but there is broad overlap in the data ranges (Emberizidae/Passerellidae = 1.5–624.1 Hz/ms; Tyrannidae = 1.4–441.7 Hz/ms) (box plots show 1st, 2nd, and 3rd quartiles and whiskers at maximum of 1.5 * IQR). Data points reflect ranges for each species included in the analysis. (b) While the distributions differ statistically (ANOVA: *F* = 13.42, *df *= 1,304, *p* = .00026), maximal up‐sweep FM rates and maximal down‐sweep FM rates (Hz/ms) are similar in range in the two families (means: 203.04 and 156.06, ranges: 0–1,872.5 Hz and 0–3,335.5 Hz, respectively). Data points represent individual syllables in the respective data sets. One tyrannid data point was of a much higher value than the bulk of the data and was omitted from the plot for improved visualization of distributions, but this data point was included in analyses, as we did not determine that it was an outlier. (c) While the distributions differ statistically (NOVA: *F* = 85.89, *df *= 1,304, *p *= <0.00001), maximal down‐sweep FM rates are similar in range in the two families (means: −250.23 and −132.80; ranges: −2,220.6 to 0.0 and −1,230.3 to 0.0 Hz/ms, respectively). Interestingly, both the up‐sweep rates (muscle effort and/or increased pressure) and down‐sweep rates (more likely passive recoil) show similar magnitudes. Data points are values for individual syllables

To compare the potential for FM in each family, we calculated the mean of the maximal FM rates of all syllables for each species. Despite the two different mechanisms for controlling frequency, species in both families achieve similar maximal FM rates (Figure 1a). Whereas mean FM rate is significantly higher in Emberizidae/Passerellidae than in Tyrannidae, the difference between the means is small compared with the ranges found in both family groups. Similarly, the maximum rate for frequency up‐sweeps (Figure 1b) and down‐sweeps (Figure 1c) is higher in the emberizid/passerellid species, but both frequency control mechanisms achieve similar ranges.

**FIGURE 2:**
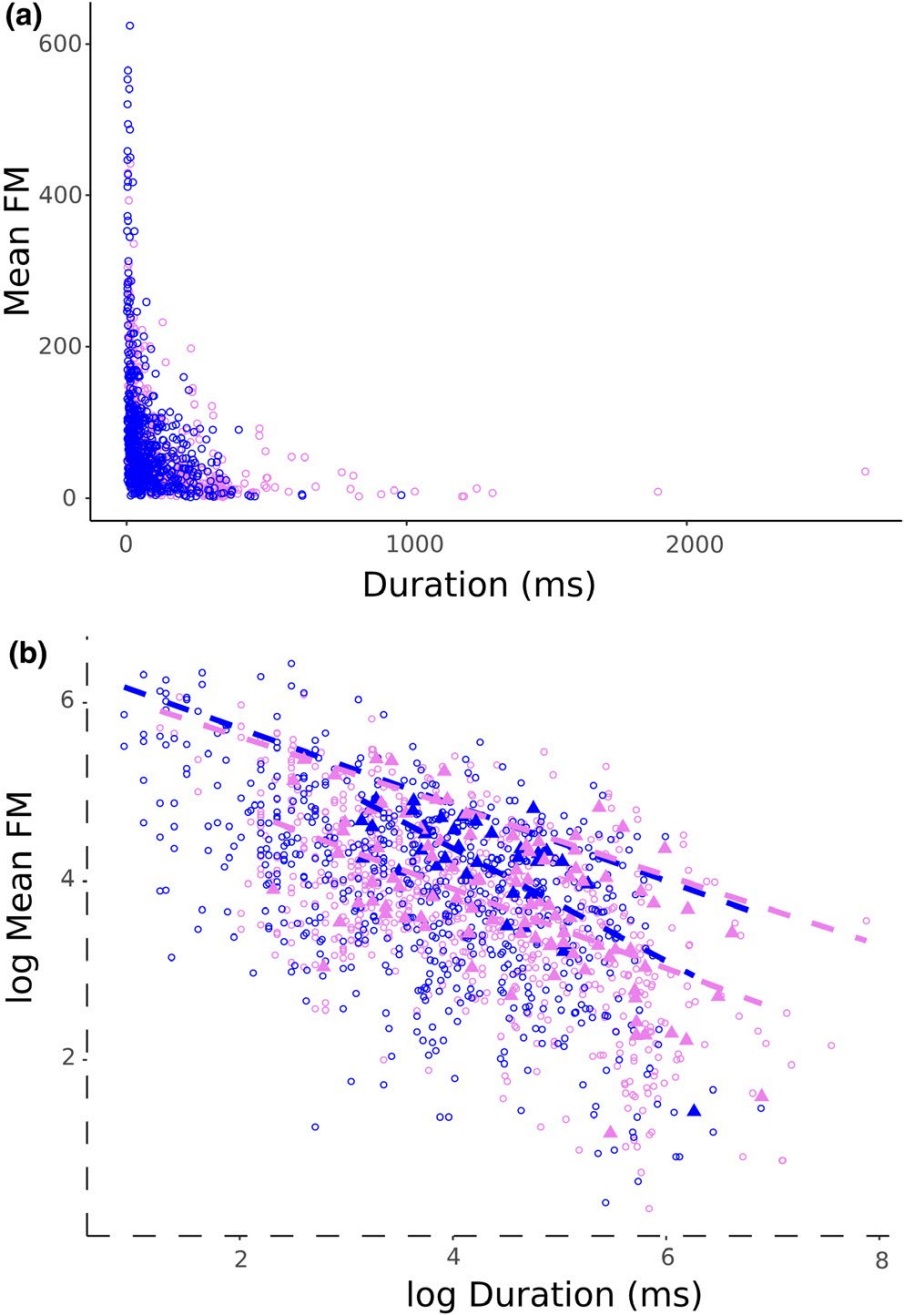
(a) Syllable‐level mean FM and duration data show a bounded relationship. (b) 90% quantile regressions (dashed lines) of natural log‐transformed syllable‐level data (open circles) show similar dependence of frequency modulation on duration (frequency modulation decreases with increasing duration; interaction *p* = .54; slopes = −0.39, −0.43), indicating that the upper bound for production of FM over time is limited in the same way between the two groups. However, the slopes of the phylogenetically controlled GLS regressions (solid lines) of species mean values (filled triangles) are different (interaction *p* = .028; slopes = −0.63, −0.44). Observing the intersection of the PGLS regression lines at longer durations suggests that the emberizid/passerellid species may be able to achieve higher FM at short syllables, but that FM production with long durations is similarly limited between the two groups. Emberizidae/Passerellidae, blue; Tyrannidae, violet; shaded regions represent 95% confidence regions for PGLS regressions

Next, we asked whether the two frequency control mechanisms differ in how long a high FM rate can be sustained over the course of a syllable. FM rate and duration show a bounded relationship in both groups (Figure 2a). The log‐transformed data show a decline in mean FM rate with increasing duration (Figure 2b). There was little difference between the two family groups in the slopes of the 90th quantile regression (interaction *p* = .54; slopes = −0.39, −0.43), suggesting that the frequency control mechanisms of both groups appear to be similarly limited in how long FM can be sustained. Controlling for the effects of phylogenetic relationships, PGLS regressions of log‐transformed species mean FM rates and durations show different slopes between family groups (interaction *p* = .028; slopes = −0.63, −0.44). These analyses suggest that the emberizid/passerellid species may be able to produce on average higher FM at shorter durations than the tyrannids, but the two groups produce similar FM at longer durations (Figure 2b).

**FIGURE 3:**
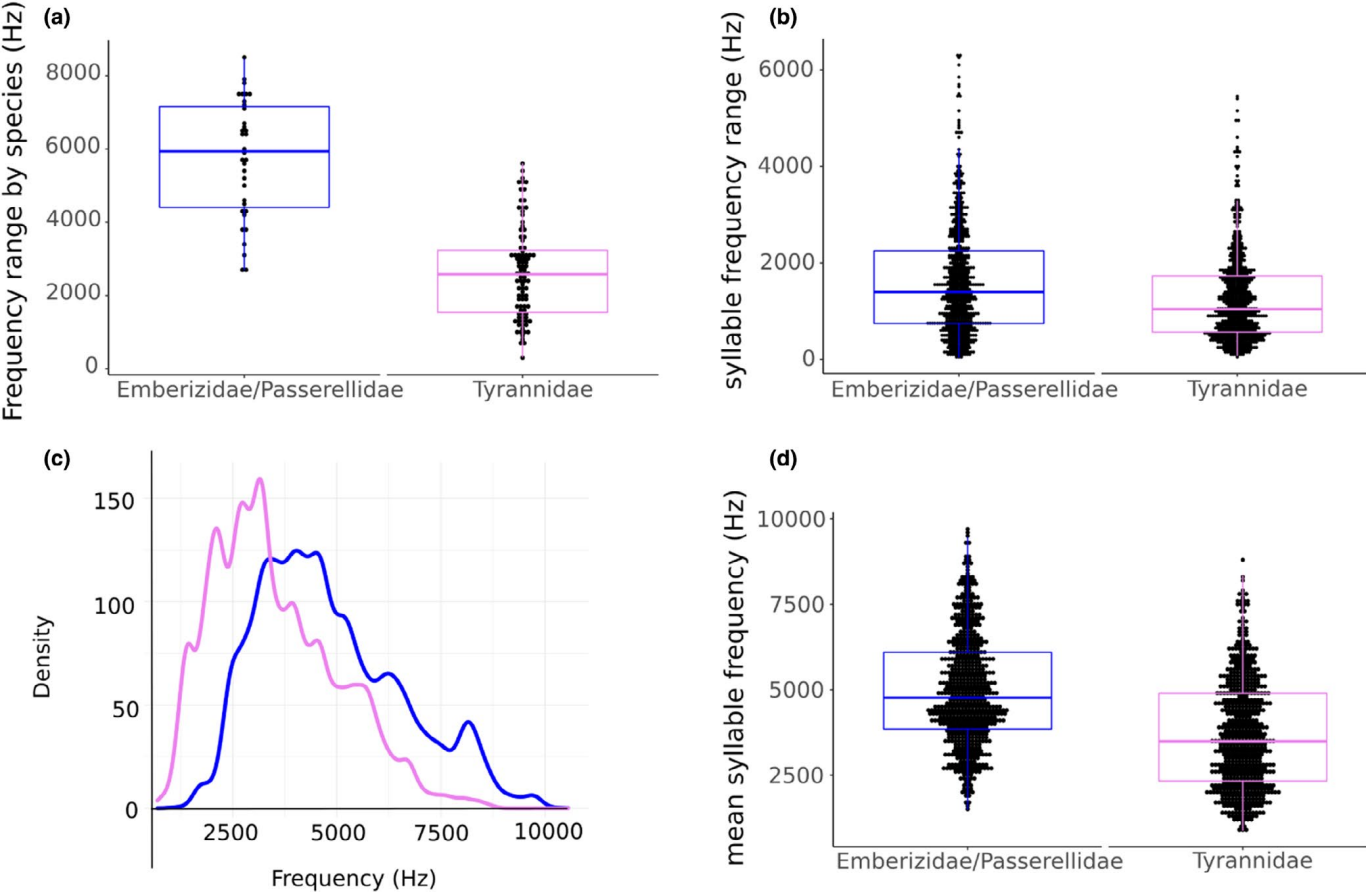
(a) The frequency ranges of individual species are markedly greater in the oscine group than in the suboscine group (means: 5,742.36 and 2,628.35, respectively; Welch's two‐sample *t* test: *t* = 10.27, *df *= 50.30, *p* < .00001). (b) Frequency ranges of individual syllables for the oscines and suboscine groups. The group ranges show a high degree of overlap. Means: 1,636.67 and 1,271.66 Hz, ranges: 30.33–6,318.59 Hz and 69.96–5,456.33 Hz, respectively. Welch's two‐sample *t* test: *t* = 6.20, *df *= 1,230, *p*‐value = 7.555e−10. (c) The distribution of all frequency measurements shows a shift toward higher frequencies in the emberizids/passerellids (blue) as compared to that of tyrannids (pink), but both are similarly skewed. (d) Mean frequencies of individual syllables. Welch's two‐sample *t* test *t* = 14.40, *df *= 1,304, *p* < .00001

In light of the small difference between the two family groups despite the different frequency control mechanisms, we compared the frequency ranges of song repertoires for species in each family group. Examples show that emberizid/passerellid species can display very broad frequency ranges, but some species also sing with a much more limited range (Figure 3). Tyrannids tend to have more narrow frequency ranges, although some also achieve remarkable breadth. The comparison between the groups yields a clear, significant difference in mean frequency range (Figure 3a). Notably, the mean frequency range of emberizid/passerellid species is approximately 3,100 Hz greater than that of tyrannids.

To see whether this difference is attributable to larger syllable repertoires of more diverse frequency ranges or to larger frequency ranges of individual syllables, we compared the frequency ranges of individual syllables between the two family groups. Although there is a highly significant difference in the mean frequency range (Figure 3b), the data ranges for both groups overlap broadly. The distribution of all frequency measurements also shows this difference, but otherwise the distributions are similar (Figure 3c). The difference between means is only 400 Hz, which cannot explain the large difference in repertoire frequency range.

Finally, we compared the use of frequency space by species in the two family groups. Emberizid/passerellid species tend to have a higher mean frequency than tyrannid species (Figure 3d), but both groups collectively occupy a broad frequency range (Figure 3b), suggesting that the difference in frequency control mechanisms does not limit either group in the range of fundamental frequencies that can be generated.

**FIGURE 4:**
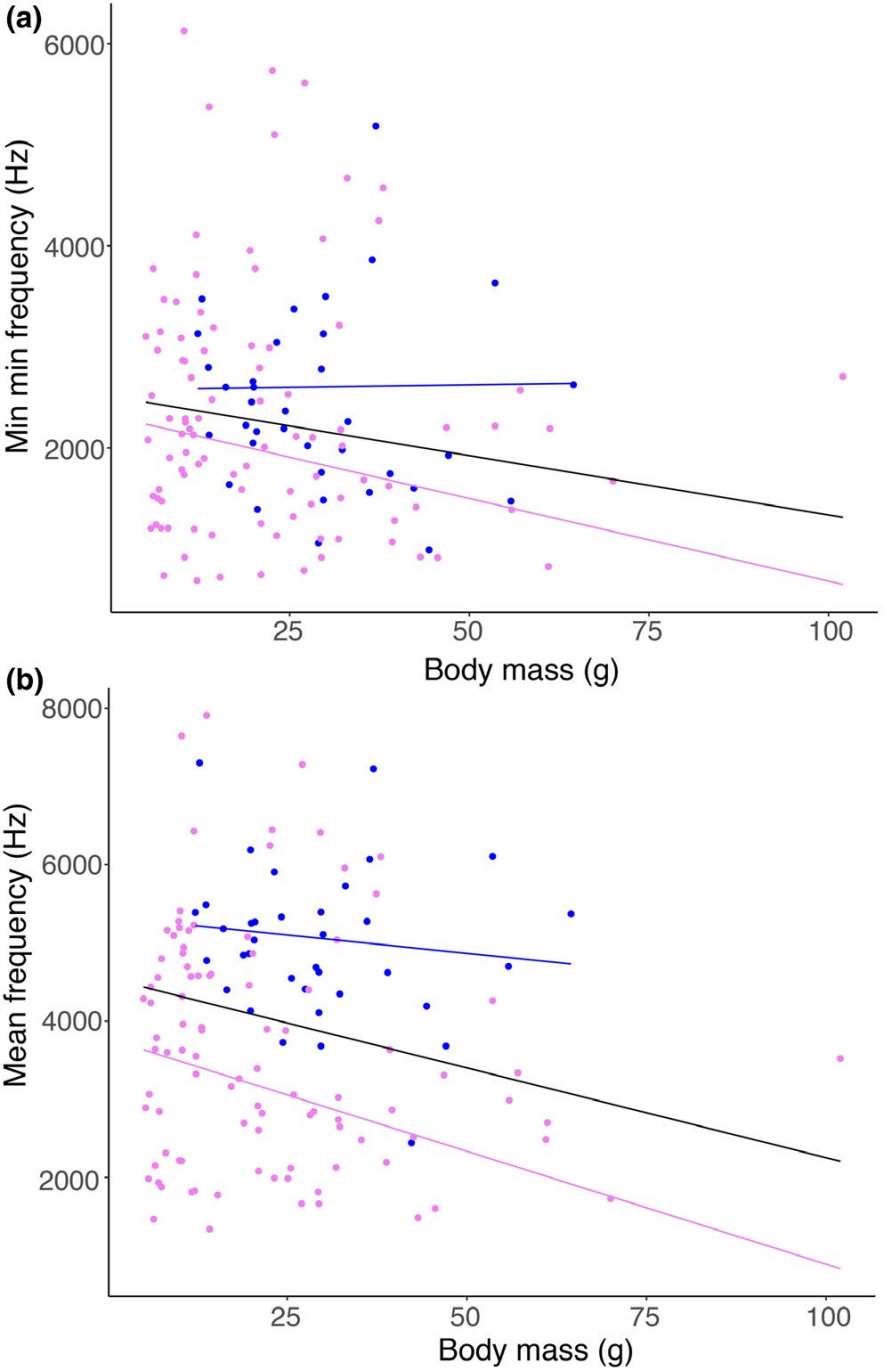
(a) Body mass and species minima of minimum frequency of syllables. Suboscines show a negative relationship between body mass and minimum frequency, while oscines do not. Lines show phylogenetically controlled GLS regressions assuming Pagel's lambda‐controlled correlation structure for all species (black; *t *= −1.57, *df *= 133, *p* = .12), Emberizidae/Passerellidae (blue; *t* = 0.095, *df *= 36, *p* = .93), and Tyrannidae (violet; *t *= −1.69, *df *= 97, *p* = .093). (b) Body mass and species mean frequencies. Again, tyrannids show a significant negative relationship between body mass and minimum frequency, while emberizid/passerellids do not. Lines show phylogenetically controlled GLS regressions assuming Pagel's lambda‐controlled correlation structure for all species (black; *t *= −2.47, *df *= 133, *p* = .015), Emberizidae/Passerellidae (blue; *t* = 0.82, *df *= 36, *p* = .42), and Tyrannidae (violet; *t *= −2.48, *df *= 97, *p* = .015)

**FIGURE 5:**
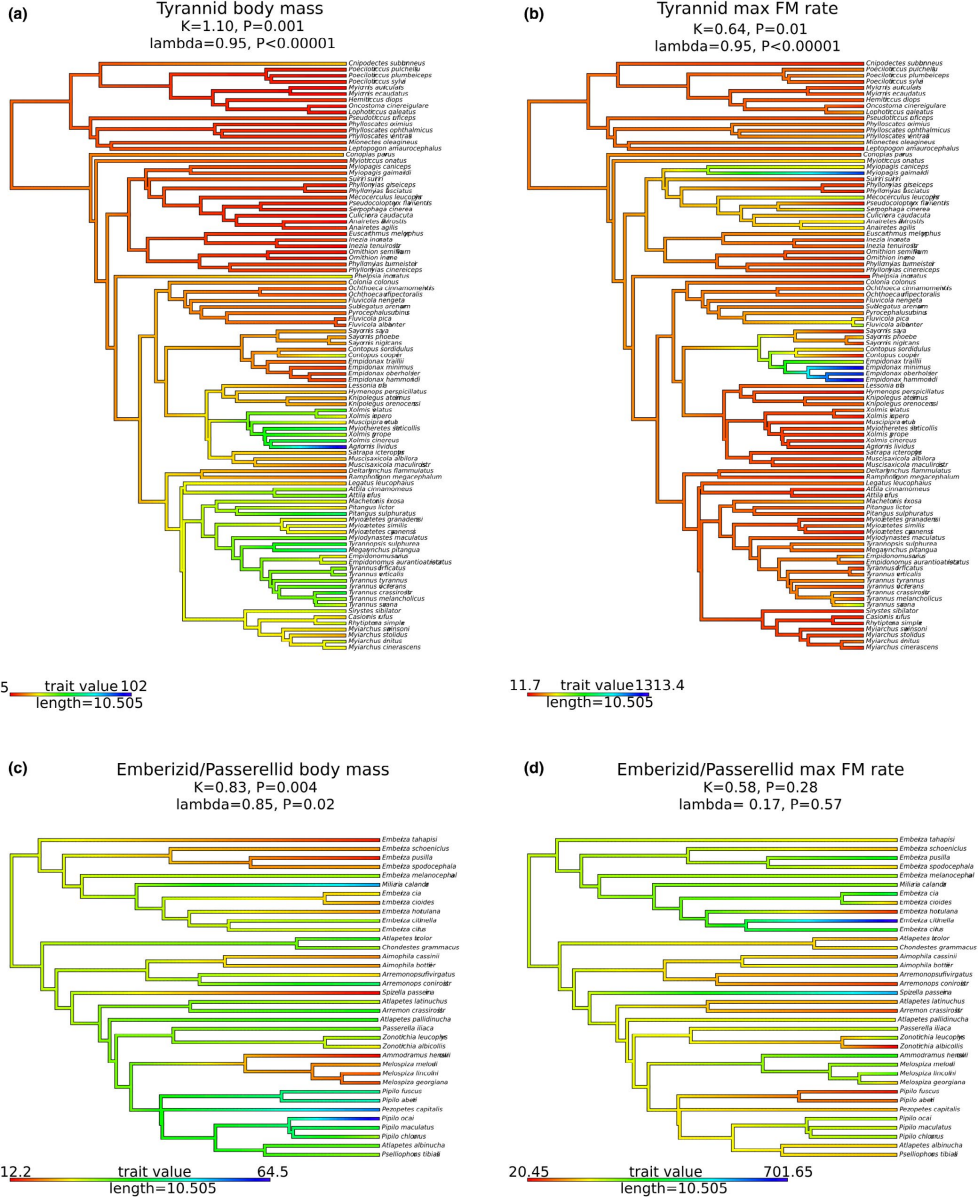
Phylogenies with continuous trait mapping for Tyrannidae (a, b) and Emberizidae/Passerellidae (c, d) groups. Traits mapped are body mass (a, c), which shows strong phylogenetic signal in both groups (closely related species are more similar in body mass than are distantly related species), and max FM rate (b, d), which shows strong phylogenetic signal in Tyrannidae (b), but not in Emberizidae/Passerellidae (d). Blomberg's *K* (Blomberg et al., 2003) and Pagel's lambda (Pagel, 1999) agree in each case. This suggests that frequency modulation is controlled by mechanisms not governed by strong genetic control in Emberizidae/Passerellidae. Colors in trees indicate trait values

Because of allometric scaling of the vibrating labia with body size (Ryan & Brenowitz, 1985; Goller & Riede, 2013), we next explored the size range of species in the two family groups and to what degree fundamental frequency is correlated with size. Mean mass for emberizid/passerellid species was slightly higher than that of tyrannids, because of a fairly large number of small‐bodied members in the latter (means ± *SD*: 29.12 ± 12.68 and 22.27 ± 16.83 g, respectively; Welch's two‐sample *t* test: *t* = 2.52, *df *= 82.79, *p* = .014) with broad overlap in the respective ranges (12.2–64.5 and 5–102 g, respectively). Tyrannid minimal and mean fundamental frequencies showed a significant decrease with increasing body mass (Figure 4b,c), whereas there was no significant relationship for emberizid/passerellids. Consistent with this result, there is not strong phylogenetic mapping for frequency parameters in emberizid/passerellids, whereas there is strong phylogenetic mapping in tyrannids (emberizid/passerellid: Pagel's lambda = 0.17, *p* = .57, Blomberg's *K* = 0.58, *p* = .28; tyrannid: Pagel's lambda = 0.95, *p* < .00001, Blomberg's *K* = 0.64, *p* = .01; Figure 5). For both groups, body mass maps significantly on phylogeny (emberizid/passerellid: Pagel's lambda = 0.85, *p* = .002, Blomberg's *K* = 0.83, *p* = .004; tyrannid: Pagel's Lambda = 0.95, *p* < .00001, Blomberg's *K* = 1.10, *p* = .001; Figure 5).

## 4 DISCUSSION

Species in the two passerine family groups included in this comparison differ in the specific mechanism by which sound frequency is controlled. Whereas driving pressure is most likely the main regulatory parameter in tyrannids, the oscine species primarily control tension of the labia with syringeal muscles (Amador et al., 2008; Döppler, Bush, Goller, et al., 2018; Goller & Riede, 2013; Goller & Suthers, 1996; Srivastava et al., 2015). Thus, FM in tyrannids is generated by modulating the air sac pressure. Denervation of the syringeal muscles does not alter FM in the same marked way as was observed in oscines (Amador et al., 2008). The intrinsic mechanical behavior of the ventilatory system in suboscines therefore must facilitate rapid changes in air sac pressure, which then cause changes in labial tension and, thus, generate FM. Remarkably, both mechanisms facilitate nearly equal ability to modulate sound frequency and the two mechanisms also enable both groups to sustain rapid FM similarly over time.

Despite the broad overlap in FM rates, direct muscular control of FM in oscines does yield slightly greater performance than pressure‐controlled modulation in suboscines. This increased ability may be relevant in regard to strong selective pressures for rapid control of acoustic features. Superfast syringeal muscles likely evolved in response to these strong selective pressures (e.g., Elemans et al., 2008; Goller, 2017). In contrast, modulation of frequency via the driving pressure is achieved by expiratory muscles (e.g., Amador et al., 2008), which do not show such rapid contraction kinetics as syringeal muscles (e.g., Srivastava et al., 2015, 2017; Suthers & Goller, 1997; Wild et al., 1998).

Rapid and broad modulation of frequency in oscines has been identified as a potential song feature that may be under strong selection (e.g., Podos, 1997; Geberzahn & Aubin, 2014; for review, e.g., Podos & Sung, 2020). The limiting motor mechanism arises in the contraction kinetics of syringeal muscles (Döppler, Bush, Amador, et al., 2018; Döppler, Bush, Goller, et al., 2018; Elemans et al., 2008; Uchida et al., 2010). In suboscines, selective forces for rapid and broad FM must act on the respiratory muscles, but it is not known whether or not expiratory muscles are specialized in suboscine species, which generate song syllables with very rapid FM. A trade‐off between frequency bandwidth and syllable repetition rate has also been documented for suboscine taxa, but it is unclear how strong selective forces are on this trait (e.g., Derryberry et al., 2012). The rate‐limiting motor systems must be the motor system that generates the modulation (i.e., the respiratory muscles in suboscines and syringeal muscles in oscines), but correlative evidence suggested a limit on upper vocal tract filtering in multispecies comparisons (e.g., Derryberry et al., 2012; García & Tubaro, 2018; Huber & Podos, 2006; Podos, 1997, 2001) and some, but not all, within‐species comparisons (e.g., Badyaev et al., 2008; Lu et al., 2014; Slabbekoorn & Smith, 2000). Irrespective of the precise selective dynamics, in both suboscines and oscines, the ability to generate very rapid frequency sweeps appears to be important in some species.

The finding that both frequency control mechanisms can give rise to individual syllables with broad frequency range and rapid modulation rates merits further study of the underlying motor mechanisms. Additionally, the main difference between suboscines and oscines in this study, the expanded spectral range of vocal repertoires in oscines, requires possible explanations. An obvious possibility is that ecological conditions between the compared species are sufficiently different to exert differential selective forces on frequency parameters of song. We do not see an ecological driving force as the main explanation for the observed differences. To minimize the possibility of a major influence of habitat conditions, the selected species of both groups occur in a wide range of different habitats from tropics to temperate zones, and many tyrannid and passerellid species sampled here inhabit the same habitat types. Nevertheless, we cannot totally exclude the possibility that some contribution from natural selective forces arising from habitat‐specific sound transmission properties may help explain the observed differences (see also below).

A second and strong possibility is that the observed differences reflect the respective song development in suboscines and oscines (e.g., Gahr, 2000; Jarvis, 2004). Tyrannids develop song innately and are not vocal learners (Kroodsma, 1984, 1989; Kroodsma & Konishi, 1991), whereas almost all oscines appear to rely on learning for at least some aspects of their song (e.g., Hultsch & Todt, 2008; Kroodsma, 1983; Love et al., 2019). The striking difference in frequency ranges (means differ by 3,100 Hz) of the entire song syllable repertoire (Figure 3a) between tyrannids and emberizids/passerellids may be explained by oscine vocal learning. Vocal learning may facilitate more independent use of the two sound generators. In oscines, the two sound generators can be tuned to different frequencies (e.g., Prince et al., 2011) and can be used in manifold ways to enhance spectral features of song. For example, each sound source can be used unilaterally to give rise to sounds with different frequency in a sequential and concatenated manner, or the two sets of labia can be used simultaneously to give rise to two independent frequencies (for reviews, e.g., Goller, 2017; Suthers & Zollinger, 2008). It is not known whether nonvocal learners such as tyrannids also have such independent control over the two sound sources, but our data suggest that this is not the case. Differently tuned sound generators can be used independently and muscular control of each source contributes to an expansion of the frequency range. In addition, drastically increased neural space for vocal control (e.g., DeVoogd et al., 1993; Moore et al., 2011; Nowicki & Searcy, 2014) enables vocal learners to generate a larger number of different syllables. Together, these factors allow individual oscine species to cover a broader frequency range within their song repertoires.

While the ability to independently use the two sound generators is likely to explain the difference in mean spectral range of individual syllables between the two groups, it is noteworthy that the difference in the means (365 Hz) is only small (Figure 3b). This small tendency for increased syllable frequency range in oscines suggests that many species do not make use of concatenation of left–right syringeal contributions (e.g., Suthers, 1999; Suthers & Zollinger, 2008) to increase the range of frequency sweeps. The expansion of frequency range of individual syllables is only one of many performance criteria and, evidently, does not universally constitute an important salient song characteristic.

Consistent with these interpretations are the findings for how frequency measures relate to body mass in the two groups. The lack of a significant negative relationship between body size and minimal or mean sound frequency for the emberizid/passerellid species could be the result of the ability to exert neural control over sound frequency. Neural control, combined with morphological specialization of the labia (e.g., Riede & Goller, 2014), allows emberizids/passerellids to achieve more independence from physical aspects such as total labial mass, than is possible in tyrannids. However, comparative information on the labial design in oscines is limited to only a few species, and no comparison is possible with Tyrannidae. It is therefore not possible to specifically assess to what degree labial design, rather than neural control, can attribute to the differences in this study. The fact that body mass significantly maps on both phylogenies indicates that these findings are not an artifact of different relatedness of the sampled taxa within each family group or the different sample sizes. In an analysis of a large data set, a significant negative relationship between body size and frequency was found for both oscines and suboscines with similar slopes, explaining nearly a third of the variation (Pearse et al., 2018). The larger range of body sizes and increased statistical power may explain why we did not find a significant relationship in emberizids/passerellids, but it is possible that smaller taxonomic groups may not exhibit this relationship for the reasons discussed above.

A larger evolutionary question to be addressed by these data was whether or not the different mechanisms used to control sound frequency led to a different exploitation of frequency space by both rapidly radiating clades. While this comparison includes a limited number of suboscine and oscine species, the results point toward some more general conclusions and indicate certain evolutionary patterns. The data strongly indicate that species in both family groups have evolved songs that cover a similar frequency range. The main difference is a slight shift toward lower frequencies in tyrannids, but the general range of syllable frequencies overlaps very broadly (Figure 3b). Basically, species in both groups exploit a range defined at the low end by the ability to generate low frequencies (Jensen et al., 2007) and at high frequencies by the upper range of passerine hearing (e.g., Dooling et al., 2000). Whereas individual emberizid/passerellid species occupy broader frequency ranges than tyrannid species, as groups they make use of similar ranges. This suggests that natural selective forces drove occupation of available frequency space in both groups. Such a view is consistent with avoidance of masking sounds in the environment, arising from abiotic sources or other sound‐generating organisms, such as insects, frogs, birds, and mammals (sensory drive—e.g., Tobias et al., 2010).

Reflecting back on the differences in production mechanisms, we can predict two possible reasons for the broad range of frequencies within the family Tyrannidae. In the absence of elaborate neural control by syringeal muscles, the different frequency ranges of tyrannid vocal repertoires must either be the result of size differences in the sound‐generating labia or be the result of differences in the nonlinearity of transferring pressure changes into frequency variation between species. Within oscines, tensing of the labia by syringeal muscles allows the production of higher frequencies, thus predicting less morphological variation of the labia. Vocal learning facilitates the evolution of broad and diverse acoustic features. While morphological specialization can achieve the same acoustic features, it limits the breadth of the features one specialized syrinx can generate (Garcia et al., 2017). The stronger relationship between frequency and body mass in tyrannids is consistent with this prediction.

## CONFLICT OF INTEREST

The authors declare no conflict of interest.

## AUTHOR CONTRIBUTIONS

**Franz Goller:** Conceptualization (equal); data curation (equal); formal analysis (equal); investigation (equal); methodology (equal); project administration (equal); validation (equal); visualization (equal); writing‐original draft (equal); writing‐review & editing (equal). **Jay Love:** Conceptualization (equal); formal analysis (equal); methodology (equal); software (equal); visualization (equal); writing‐original draft (equal); writing‐review & editing (equal). **Gabriel Mindlin:** Conceptualization (equal); methodology (equal); validation (equal); visualization (equal); writing‐original draft (equal); writing‐review & editing (equal).

## Supporting information

Appendix S1

## Data Availability

Data available via Dryad: https://doi.org/10.5061/dryad.0vt4b8gz9.
