# Supplementary material for: Different frequency control mechanisms and the exploitation of frequency space in passerines
Source: Ecol Evol. 2021 Apr 7;11(11):6569–78. doi: 10.1002/ece3.7510 (PMC8207358; doi:10.1002/ece3.7510)
Supplement: Supplementary file 1 — Appendix S1 [file ECE3-11-6569-s001.docx]

**Appendix I.**

| **Number** | **Species** | **Family** | **Body mass (g)** |
| --- | --- | --- | --- |
| 1 | Agriornis_lividus | tyrannid | 102 |
| 2 | Aimophila_botterii | emberizid/passerellid | 19.9 |
| 3 | Aimophila_cassinii | emberizid/passerellid | 18.9 |
| 4 | Ammodramus_henslowii | emberizid/passerellid | 12.8 |
| 5 | Anairetes_agilis | tyrannid | 10.1 |
| 6 | Anairetes_flavirostris | tyrannid | 6 |
| 7 | Arremon_crassirostris | emberizid/passerellid | 37 |
| 8 | Arremonops_conirostris | emberizid/passerellid | 42.3 |
| 9 | Arremonops_rufivirgatus | emberizid/passerellid | 23.2 |
| 10 | Atlapetes_albinucha | emberizid/passerellid | 33.1 |
| 11 | Atlapetes_latinuchus | emberizid/passerellid | 27.5 |
| 12 | Atlapetes_pallidinucha | emberizid/passerellid | 36.1 |
| 13 | Atlapetes_tricolor | emberizid/passerellid | 36.5 |
| 14 | Attila_cinnamomeus | tyrannid | 38.8 |
| 15 | Attila_rufus | tyrannid | 42.6 |
| 16 | Casiornis_rufus | tyrannid | 24.8 |
| 17 | Chondestes_grammacus | emberizid/passerellid | 29 |
| 18 | Cnipodectes_subbrunneus | tyrannid | 23.2 |
| 19 | Colonia_colonus | tyrannid | 18.3 |
| 20 | Conopias_parvus | tyrannid | 21 |
| 21 | Contopus_cooperi | tyrannid | 32.1 |
| 22 | Contopus_sordidulus | tyrannid | 13.1 |
| 23 | Culicivora_caudacuta | tyrannid | 5.8 |
| 24 | Deltarhynchus_flammulatus | tyrannid | 17.2 |
| 25 | Emberiza_cia | emberizid/passerellid | 24.2 |
| 26 | Emberiza_cioides | emberizid/passerellid | 20 |
| 27 | Emberiza_cirlus | emberizid/passerellid | 25.6 |
| 28 | Emberiza_citrinella | emberizid/passerellid | 29.7 |
| 29 | Emberiza_hortulana | emberizid/passerellid | 19.9 |
| 30 | Emberiza_melanocephala | emberizid/passerellid | 29.7 |
| 31 | Emberiza_pusilla | emberizid/passerellid | 13.8 |
| 32 | Emberiza_schoeniclus | emberizid/passerellid | 19.7 |
| 33 | Emberiza_spodocephala | emberizid/passerellid | 20.4 |
| 34 | Emberiza_tahapisi | emberizid/passerellid | 13.7 |
| 35 | Empidonax_hammondii | tyrannid | 10.6 |
| 36 | Empidonax_minimus | tyrannid | 10 |
| 37 | Empidonax_oberholseri | tyrannid | 10.4 |
| 38 | Empidonax_traillii | tyrannid | 13.1 |
| 39 | Empidonomus_aurantioatrocristatus | tyrannid | 33 |
| 40 | Empidonomus_varius | tyrannid | 27.1 |
| 41 | Euscarthmus_meloryphus | tyrannid | 6.8 |
| 42 | Fluvicola_albiventer | tyrannid | 11.6 |
| 43 | Fluvicola_nengeta | tyrannid | 21 |
| 44 | Fluvicola_pica | tyrannid | 12.3 |
| 45 | Hemitriccus_diops | tyrannid | 10 |
| 46 | Hymenops_perspicillatus | tyrannid | 22.9 |
| 47 | Inezia_inornata | tyrannid | 12 |
| 48 | Inezia_tenuirostris | tyrannid | 5 |
| 49 | Knipolegus_aterrimus | tyrannid | 20.21 |
| 50 | Knipolegus_orenocensis | tyrannid | 19 |
| 51 | Legatus_leucophaius | tyrannid | 22.2 |
| 52 | Leptopogon_amaurocephalus | tyrannid | 11.7 |
| 53 | Lessonia_rufa | tyrannid | 13.8 |
| 54 | Lophotriccus_galeatus | tyrannid | 6.6 |
| 55 | Machetornis_rixosa | tyrannid | 29.6 |
| 56 | Mecocerculus_leucophrys | tyrannid | 10.4 |
| 57 | Megarynchus_pitangua | tyrannid | 70 |
| 58 | Melospiza_georgiana | emberizid/passerellid | 16.1 |
| 59 | Melospiza_lincolnii | emberizid/passerellid | 16.6 |
| 60 | Melospiza_melodia | emberizid/passerellid | 20.5 |
| 61 | Miliaria_calandra | emberizid/passerellid | 53.6 |
| 62 | Mionectes_oleagineus | tyrannid | 12.1 |
| 63 | Muscipipra_vetula | tyrannid | 27 |
| 64 | Muscisaxicola_albilora | tyrannid | 22.6 |
| 65 | Muscisaxicola_maculirostris | tyrannid | 14.2 |
| 66 | Myiarchus_cinerascens | tyrannid | 28.2 |
| 67 | Myiarchus_crinitus | tyrannid | 32.1 |
| 68 | Myiarchus_stolidus | tyrannid | 20.8 |
| 69 | Myiarchus_swainsoni | tyrannid | 25.1 |
| 70 | Myiodynastes_maculatus | tyrannid | 43.2 |
| 71 | Myiopagis_caniceps | tyrannid | 10.5 |
| 72 | Myiopagis_gaimardii | tyrannid | 12.6 |
| 73 | Myiornis_auricularis | tyrannid | 5.3 |
| 74 | Myiornis_ecaudatus | tyrannid | 6 |
| 75 | Myiotheretes_striaticollis | tyrannid | 61.2 |
| 76 | Myiotriccus_ornatus | tyrannid | 9.9 |
| 77 | Myiozetetes_cayanensis | tyrannid | 25.9 |
| 78 | Myiozetetes_granadensis | tyrannid | 29.3 |
| 79 | Myiozetetes_similis | tyrannid | 28 |
| 80 | Ochthoeca_cinnamomeiventris | tyrannid | 12 |
| 81 | Ochthoeca_rufipectoralis | tyrannid | 10.5 |
| 82 | Oncostoma_cinereigulare | tyrannid | 6.4 |
| 83 | Ornithion_inerme | tyrannid | 7 |
| 84 | Ornithion_semiflavum | tyrannid | 6.6 |
| 85 | Passerella_iliaca | emberizid/passerellid | 32.3 |
| 86 | Pezopetes_capitalis | emberizid/passerellid | 55.8 |
| 87 | Phelpsia_inornatus | tyrannid | 29.4 |
| 88 | Phyllomyias_burmeisteri | tyrannid | 11.1 |
| 89 | Phyllomyias_cinereiceps | tyrannid | 10.3 |
| 90 | Phyllomyias_fasciatus | tyrannid | 10.3 |
| 91 | Phyllomyias_griseiceps | tyrannid | 7.2 |
| 92 | Phylloscartes_eximius | tyrannid | 7.5 |
| 93 | Phylloscartes_ophthalmicus | tyrannid | 9.2 |
| 94 | Phylloscartes_ventralis | tyrannid | 8.3 |
| 95 | Pipilo_aberti | emberizid/passerellid | 47.1 |
| 96 | Pipilo_chlorurus | emberizid/passerellid | 29.4 |
| 97 | Pipilo_fuscus | emberizid/passerellid | 44.4 |
| 98 | Pipilo_maculatus | emberizid/passerellid | 39 |
| 99 | Pipilo_ocai | emberizid/passerellid | 64.5 |
| 100 | Pitangus_lictor | tyrannid | 25.5 |
| 101 | Pitangus_sulphuratus | tyrannid | 61 |
| 102 | Poecilotriccus_plumbeiceps | tyrannid | 5.7 |
| 103 | Poecilotriccus_pulchellus | tyrannid | 8.1 |
| 104 | Poecilotriccus_sylvia | tyrannid | 7.1 |
| 105 | Pselliophorus_tibialis | emberizid/passerellid | 30 |
| 106 | Pseudocolopteryx_flaviventris | tyrannid | 7.5 |
| 107 | Pseudotriccus_ruficeps | tyrannid | 11.3 |
| 108 | Pyrocephalus_rubinus | tyrannid | 14.4 |
| 109 | Ramphotrigon_megacephalum | tyrannid | 14.2 |
| 110 | Rhytipterna_simplex | tyrannid | 31.8 |
| 111 | Satrapa_icterophrys | tyrannid | 21.5 |
| 112 | Sayornis_nigricans | tyrannid | 19.5 |
| 113 | Sayornis_phoebe | tyrannid | 19.7 |
| 114 | Sayornis_saya | tyrannid | 20.9 |
| 115 | Serpophaga_cinerea | tyrannid | 8.3 |
| 116 | Sirystes_sibilator | tyrannid | 32.3 |
| 117 | Spizella_passerina | emberizid/passerellid | 12.2 |
| 118 | Sublegatus_arenarum | tyrannid | 12.3 |
| 119 | Suiriri_suiriri | tyrannid | 15.3 |
| 120 | Tyrannopsis_sulphurea | tyrannid | 53.6 |
| 121 | Tyrannus_crassirostris | tyrannid | 55.9 |
| 122 | Tyrannus_forficatus | tyrannid | 39.3 |
| 123 | Tyrannus_melancholicus | tyrannid | 37.4 |
| 124 | Tyrannus_savana | tyrannid | 31.9 |
| 125 | Tyrannus_tyrannus | tyrannid | 38 |
| 126 | Tyrannus_verticalis | tyrannid | 39.6 |
| 127 | Tyrannus_vociferans | tyrannid | 45.6 |
| 128 | Xolmis_cinereus | tyrannid | 57.1 |
| 129 | Xolmis_irupero | tyrannid | 28.7 |
| 130 | Xolmis_pyrope | tyrannid | 35.3 |
| 131 | Xolmis_velatus | tyrannid | 46.8 |
| 132 | Zonotrichia_albicollis | emberizid/passerellid | 24.4 |
| 133 | Zonotrichia_leucophrys | emberizid/passerellid | 29.4 |
